# Transcriptome and methylome profiling reveals relics of genome dominance in the mesopolyploid *Brassica oleracea*

**DOI:** 10.1186/gb-2014-15-6-r77

**Published:** 2014-06-10

**Authors:** Isobel AP Parkin, Chushin Koh, Haibao Tang, Stephen J Robinson, Sateesh Kagale, Wayne E Clarke, Chris D Town, John Nixon, Vivek Krishnakumar, Shelby L Bidwell, France Denoeud, Harry Belcram, Matthew G Links, Jérémy Just, Carling Clarke, Tricia Bender, Terry Huebert, Annaliese S Mason, J Chris Pires, Guy Barker, Jonathan Moore, Peter G Walley, Sahana Manoli, Jacqueline Batley, David Edwards, Matthew N Nelson, Xiyin Wang, Andrew H Paterson, Graham King, Ian Bancroft, Boulos Chalhoub, Andrew G Sharpe

**Affiliations:** 1 Agriculture and Agri-Food Canada, 107 Science Place, Saskatoon, S7N0X2, Canada; 2 National Research Council Canada, 110 Gymnasium Place, Saskatoon S7N0W9, Canada; 3 J. Craig Venter Institute, 9704 Medical Center Drive, Rockville, MD 20850, USA; 4 Commissariat à l’Energie Atomique (CEA), Institut de Génomique, BP5706, Evry, 91057, France; 5 Centre National de Recherche Scientifique (CNRS), UMR 8030, CP5706 Evry, France; 6 Université d’Evry, UMR 8030, Evry, CP5706, France; 7 Organization and Evolution of Plant Genomes, Unité de Recherche en Génomique Végétale, Unité Mixte de Rechercheé 1165, (Inland Northwest Research Alliance Institut National de Recherche Agronomique - Centre National de la Recherche Scientifique, Université Evry Val d’Essonne), Evry, France; 8 School of Agriculture and Food Sciences, The University of Queensland, Brisbane 4072, Australia; 9 Division of Biological Sciences, Bond Life Sciences Center, University of Missouri, Columbia, MO 65211-7310, USA; 10 Warwick Life Sciences, The University of Warwick, Warwick CV35 9EF, UK; 11 School of Plant Biology/ The UWA Institute of Agriculture, The University of Western Australia, 35 Stirling Highway, Crawley, WA 6009, Australia; 12 Plant Genome Mapping Laboratory, University of Georgia, Athens GA 30602, USA; 13 Southern Cross Plant Science, Southern Cross University, Lismore, NSW 2480, Australia; 14 Centre for Novel Agricultural Products (CNAP), Department of Biology, University of York, Wentworth Way, Heslington, York YO10 5DD, UK

## Abstract

**Background:**

*Brassica oleracea* is a valuable vegetable species that has contributed to human health and nutrition for hundreds of years and comprises multiple distinct cultivar groups with diverse morphological and phytochemical attributes. In addition to this phenotypic wealth, *B. oleracea* offers unique insights into polyploid evolution, as it results from multiple ancestral polyploidy events and a final Brassiceae-specific triplication event. Further, *B. oleracea* represents one of the diploid genomes that formed the economically important allopolyploid oilseed, *Brassica napus*. A deeper understanding of *B. oleracea* genome architecture provides a foundation for crop improvement strategies throughout the *Brassica* genus.

**Results:**

We generate an assembly representing 75% of the predicted *B. oleracea* genome using a hybrid Illumina/Roche 454 approach. Two dense genetic maps are generated to anchor almost 92% of the assembled scaffolds to nine pseudo-chromosomes. Over 50,000 genes are annotated and 40% of the genome predicted to be repetitive, thus contributing to the increased genome size of *B. oleracea* compared to its close relative *B. rapa*. A snapshot of both the leaf transcriptome and methylome allows comparisons to be made across the triplicated sub-genomes, which resulted from the most recent Brassiceae-specific polyploidy event.

**Conclusions:**

Differential expression of the triplicated syntelogs and cytosine methylation levels across the sub-genomes suggest residual marks of the genome dominance that led to the current genome architecture. Although cytosine methylation does not correlate with individual gene dominance, the independent methylation patterns of triplicated copies suggest epigenetic mechanisms play a role in the functional diversification of duplicate genes.

## Background

The Brassicaceae family encompasses extensive species diversity with a wide range of intra- and inter-specific morphological and phytochemical profiles, which has contributed to their global importance in crop production. The most agriculturally significant species are found within the Brassiceae tribe where selection for targeted traits in both seed and vegetative tissues has generated high-value oilseed, vegetable and condiment crops. *Brassica oleracea* represents an important species integral to human diets that encompasses multiple cultivar groups that are classified based on the specialized morphology of their edible structures, namely kales, cabbages, broccoli, cauliflower, Brussels sprouts and kohl rabi. In addition to this radical morphological variation, the cultivars and their related wild species accumulate a range of secondary metabolites that have been implicated in promoting human health, such as compounds functioning as anti-carcinogens and anti-oxidants. *B. oleracea* (n = 9, CC genome) along with its close relative *Brassica rapa* (n = 10, AA genome) are the extant descendants of the two diploid species, which fused to form the amphidiploid *Brassica napus* (canola or oilseed rape; n = 19, AACC genome), arguably the most economically valuable species among the Brassiceae.

Brassica research has been enhanced through the recent publication of the *B. rapa* genome sequence [1]. In the current report we present the genome of the close relative *B. oleracea,* which diverged from *B. rapa* some 4 million years ago (Mya) [2]. The completion of the genome sequences of these diploid species and the anticipated assembly of the amphidiploid *B. napus* genome will provide foundational resources for not only crop improvement but also studying polyploid evolution. The importance of polyploidy as a driving force in plant speciation, diversification and evolution has been widely acknowledged [3]. The *Brassica* species carry remnants of three whole genome duplication (WGD) events, the α, β and γ events [4], the most recent of which, the α event, dates from approximately 47 Mya. In addition, the *Brassica* diploid species are mesopolyploids, having experienced an hexaploidization event approximately 23 Mya [5]. As such, *Brassica* species offer a unique opportunity to study the impacts of WGD on genome evolution, and in particular the mechanisms employed to mitigate or exploit the impact of maintaining multiple copies of each gene. Analysis of the *B. rapa* genome suggested that the hexaploidy event occurred in two discrete steps (from diploid to tetraploid, then from tetraploid to hexaploid) with a reduction in gene number at each stage, resulting in one genome (least-fractionated) copy maintaining a higher percentage of genes than the other two [6]. It has also been suggested that genome dominance has led to the inequity in the maintenance of gene copies after the WGD events, such that the least-fractionated genome of *B. rapa* exhibits higher levels of gene expression than its nuclear counterparts [7]. However, the mechanisms for maintaining such dominance are unknown, although epigenetic phenomena have been suggested as possible determinants.

The current report describes the generation of a reference genome for the mesopolyploid *B. oleracea* along with transcriptome and methylome characterization. The genome was compared with its close relative *B. rapa* to provide insights into the observed difference in genome size, with *B. oleracea* being approximately 20% larger than *B. rapa*. In addition, the legacy of the mesopolyploidy event relative to maintenance of gene copy number, relative gene expression and cytosine methylation levels was assessed.

## Results

### Genome assembly

A genome assembly of the doubled haploid *B. oleracea* kale-like type TO1000DH was generated from approximately 94× coverage of the estimated 648 Mb genome (Figure S1 in Additional file 1). The sequence data comprised previously available Sanger sequencing reads (0.01 Gb) and newly generated Illumina (56.33 Gb) and Roche/454 (0.31 Gb) sequencing data (Table S1 in Additional file 2). A hybrid assembly approach was taken using a *de Bruijn* graph-based short read assembler to generate initial contigs and scaffolds incorporating all available sequence data [8]. The software tool Bambus [9], which provides a hierarchical approach to defining contig adjacencies, was used to generate scaffolds taking advantage of large insert mate-pair libraries (8 and 17 kb) and paired bacterial artificial chromosome (BAC) end sequences. The final assembly totaling 488.6 Mb (with 9% uncalled bases) covered approximately 75% of the estimated genome size (648 Mb) and was arranged into 33,459 scaffolds (2,172 of which were greater than 2 kb), with an N50 of 850 kb (Table 1). The quality of the assembled sequence was validated through CEGMA analysis [10], reference mapping of paired-end reads to the assembly and sequence alignment with publicly available BAC sequences (Table S2 in Additional file 2; Figure S2 in Additional file 1).

**Table 1:** **Assembly statistics for *Brassica oleracea***

**Number of sequences**^**a**^	33,459	Number of bases	488,574,611
**Max length (bp)**	4,900,790		
**N50 value**	169	N50 size	850,003
**N60 value**	236	N60 size	654,695
**N70 value**	321	N70 size	488,433
**N80 value**	443	N80 size	328,342
**N90 value**	659	N90 size	142,857

^a^Data are reported for all scaffolds greater than 200 bp.

### Genetic anchoring of the genome assembly

**Figure 1:**
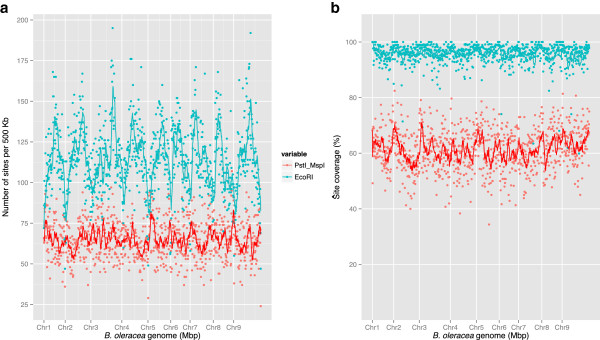
**Comparison of efficacy of GBS methods. (a)** Distribution of restriction sites across the *B. oleracea* genome, representing potential tag sites for RAD (blue) and GBS (red). **(b)** Observed tag coverage for restriction sites within the *B. oleracea* genome for RAD (blue) and GBS (red). A sliding window of 500 kb was used and the trend line is based on the mean of 10 windows.

Anchoring of the scaffolds to the linkage map of *B. oleracea* was achieved through the generation of high-density genetic maps based on two genotyping-by-sequencing (GBS) approaches. The first approach, restriction site-associated DNA (RAD) mapping, uses a single restriction enzyme (*Eco*RI) combined with shearing to capture a fraction of the genome for single nucleotide polymorphism (SNP) discovery and mapping [11]. The second GBS approach utilizes two restriction enzymes (*Pst*I and *Msp*I), one with a six-base and the second with a four-base recognition site, to effect genome reduction by capturing a subset of the genome [12]. The relative genome coverage of each method differed due to both the distribution of restriction sites and the impact of cytosine methylation (Figure 1). RAD captured a greater portion of the genome (Figure 1a) and a higher percentage of the potential sites were tagged and sequenced (Figure 1b).

RAD mapping was performed using a doubled haploid population derived from a cross between TO1000 and Early Big (var. *italica,* broccoli type), identifying 67,567 potential nucleotide variations (both SNPs and insertion/deletions or indels) segregating among the progeny. These data were compressed based on recombination breakpoints to provide 2,299 anchor points that were used to generate a high-density linkage map for *B. oleracea* (Table 2). Based on the linkage analyses, 66 scaffolds were found to be mis-assembled and were separated according to the SNP positions and available genome synteny data. In regions of low recombination the order and orientation of the scaffolds were assigned based on their syntenic relationship with *Arabidopsis thaliana* and/or *B. rapa*. The final map was confirmed through alignment of the GBS data, which provided 15,909 SNP loci that were compressed into 826 genetic bins. This allowed almost 94% of the assembly to be assigned to one of the nine pseudo-chromosomes of *B. oleracea*. In regions of reduced recombination where the scaffolds showed limited synteny with either of their Brassicaceae relatives the remaining assembled scaffolds (less than 3%) could not be accurately positioned and/or ordered relative to flanking scaffolds.

**Table 2:** **Genetic anchoring of TO1000 assembly to *B. oleracea *** **pseudochromosomes (C1 to C9)**

**Pseudo-chromosome**	**Number of linked scaffolds**	**Number of base pairs anchored**	**Percentage of base pairs anchored**	**Number of anchored scaffolds**	**Number of base pairs (%) in pseudochromosomes**
C1	154	44,537,578	9.1%	106	43,754,388 (9.0)
C2	161	53,780,918	11.0%	111	52,875,895 (10.8)
C3	142	65,831,836	13.5%	102	64,974,595 (13.5)
C4	235	55,258,765	11.3%	138	53,705,393 (11.0)
C5	141	48,366,635	9.9%	99	46,892,785 (9.6)
C6	132	40,383,462	8.3%	79	39,814,676 (8.1)
C7	129	49,639,853	10.2%	64	48,360,397 (9.9)
C8	124	43,398,395	8.9%	63	41,752,485 (8.5)
C9	192	56,144,286	11.5%	121	54,667,868 (11.2)
Total	1,410	457,341,728	93.6%	883	446,798,482 (91.5)

The total number of assembled bases was 488,535,107; the percentage genetically linked (RAD mapping) was 93.61%; the percentage anchored to pseudochromosomes was 91.46%.

### Genome annotation of *Brassica oleracea*

A combination of homology-based and *de novo* gene prediction was used to annotate 59,225 gene models in the *B. oleracea* genome [13]. Complete protein coding genes were predicted for 54,475 models. The gene models were annotated based on homology to proteins in public plant databases (Table S3 in Additional file 2). Orthologues for 94.6% (56,082) of all gene models were identified within the various UniProt databases, of which 52,774 (94%) were found in species from the Brassicaceae (Table S3 in Additional file 2). RNASeq data generated from four tissue types provided evidence of expression from 62.7% of the gene models (Table S4 in Additional file 2).

The number of *B. oleracea* gene models was 1.4-fold greater than for its close relative *B. rapa* (41,174). However, different annotation pipelines were utilized, and although repeat masking was employed, it is possible that some of the gene expansion may be due to the presence of uncharacterized transposon-related sequences [14]. Reciprocal alignments between the annotated genes of the two *Brassica* diploids using BLAST found 32,061 orthologous pairs. A further 20,343 *B. oleracea* gene models showed similarity to an annotated *B. rapa* gene, suggesting expansion of gene families or tandem duplication, and 3,507 were homologous to *B. rapa* intergenic sequence, suggesting the presence of potentially un-annotated genes in the *B. rapa* genome [1]. The remaining 3,314 genes could represent *B. oleracea*-specific genes or are possible artifacts of avaricious annotation.

**Figure 2:**
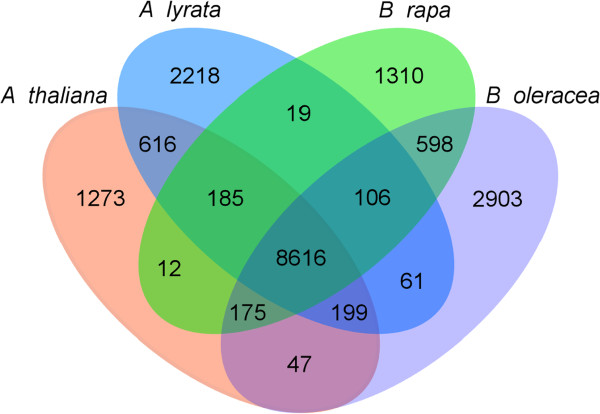
**Distribution of unique and shared gene families among Brassicaceae species.** Homologous proteins in *A. thaliana*, *A. lyrata*, *B. rapa* and *B. oleracea* were clustered into gene families using TRIBE-MCL. Numbers in individual sections indicate number of gene families (not genes).

To determine if the increase in gene number in *B. oleracea* was due to the expansion of gene families, orthologous proteins from *B. oleracea* and three completely sequenced Brassicaceae species, *A. thaliana, Arabidopsis lyrata* and *B. rapa,* were clustered into gene families based on reciprocal pairwise sequence similarities. In total, 18,338 gene families were identified, of which 8,616 were shared by all four species, 4,811 were confined to lineage II (*Brassica*) species and 4,701 to lineage I (*Arabidopsis*) species (Figure 2). About 7% (3,930 out of 59,225) of *B. oleracea* genes did not have orthologues in the other three Brassicaceae species, suggesting that these genes could potentially represent *B. oleracea*-specific orphan genes. Comparison of family-wise gene numbers from all four species revealed significant (χ^2^ test, *P* < 0.05) differences in 148 gene families (Table S5 in Additional file 2). In approximately 50% (73 out of 148) of these families the greatest expansion of gene number was observed in *B. oleracea*. Examination of predicted functions of genes belonging to these 73 families revealed an enrichment of genes involved in disease resistance and signal transduction pathways, both functional categories suggested to be prone to lineage-specific expansions in eukaryotes [15].

**Figure 3:**
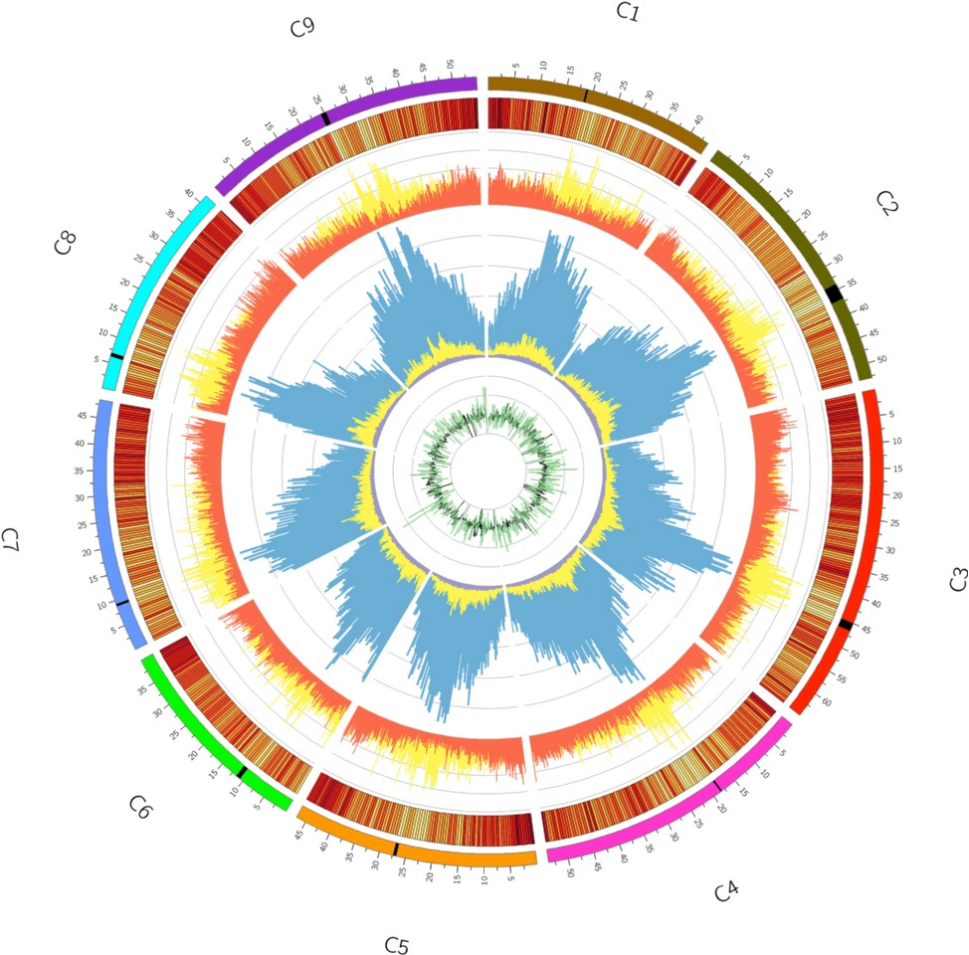
**The *Brassica oleracea *genome.** From the outside ring to the centre: 1) the nine *B. oleracea* pseudochromosomes (C1 to C9 represented on a Mb scale) are shown in different colors with putative centromeric regions indicated by black bands; 2) gene expression levels (average (log (FPKM)), bin = 500 kb), values range from 0 (yellow) to 3.19 (red); 3) the distribution of protein coding regions (nucleotides per 100 kb; orange) compared to repetitive sequences (nucleotides per 100 kb; yellow); 4) cytosine methylation levels (average number of methylated cytosines, bin = 500 kb) for mCG (blue), mCHG (yellow) and mCHH (grey); and 5) *Ka/Ks* ratios (median, bin = 500 kb) of syntenic (black) and non-syntenic (green) genes.

The distribution of repeats across the *B. oleracea* genome was as expected with a concentration of elements in proximity to the centromeric regions (Figure 3). The centromeres were positioned for each chromosome based on synteny, abundance of repetitive elements, higher methylation gradient, presence of centromere-specific marker genes [16] and half-tetrad analyses (Table S6 in Additional file 2). The *B. rapa* genome was re-annotated for repeat sequences to allow a direct comparison between the two diploid genomes, which identified some disparities in repeat content (Table 3). Although DNA transposons were over-represented in *B. oleracea* compared to *B. rapa*, the greatest difference was observed for RNA elements with retrotransposons covering over twice the base-pair space in *B. oleracea* (21.7%) than in *B. rapa* (10.3%).

**Table 3:** **Summary of repeat elements annotated in *B. rapa *** **and *B. oleracea***

	***B. rapa***			***B. oleracea***		
**Repeat element**^**a**^	**Number of copies**	**Number of base pairs**	**Percentage genome (not N)**	**Number of copies**	**Number of base pairs**	**Percentage genome (not N)**
		283,841,084	273,102,035		488,622,507	445,620,295
RLC	30,349	11,292,047	4.13%	77,899	42,075,014	9.44%
RLG	19,229	9,327,740	3.42%	52,619	36,956,399	8.29%
RLX	11,358	3,768,473	1.38%	22,357	10,165,627	2.28%
RSX	4,248	549,493	0.20%	8,442	1,141,202	0.26%
RIL	2,658	1,200,998	0.44%	4,845	2,500,079	0.56%
RIX	4,432	2,059,525	0.75%	6,453	4,002,179	0.90%
Subtotal			**10.33%**			**21.73%**
DTA	14,722	2,915,918	1.07%	22,838	5,053,940	1.13%
DTC	17,742	5,289,213	1.94%	44,958	16,124,758	3.62%
DTH	2,057	581,852	0.21%	4,028	1,193,462	0.27%
DTM	13,307	3,022,686	1.11%	19,208	5,098,147	1.14%
DTT	16,867	2,720,339	1.00%	33,537	6,425,309	1.44%
DTX	29,919	7,453,903	2.73%	46,687	12,566,497	2.82%
DHH	46,182	10,206,949	3.74%	67,720	18,514,526	4.15%
Subtotal			**11.79%**			**14.58%**
Unclassified		2,183,677	0.80%		3,945,235	0.89%
Total	**213,070**	**62,572,813**	**22.91%**	**411,591**	**165,762,374**	**37.20%**

^a^Repeat elements are named according to Wicker *et al*. [44].

### Comparative genome organization of *B. oleracea* with close relatives

**Figure 4:**
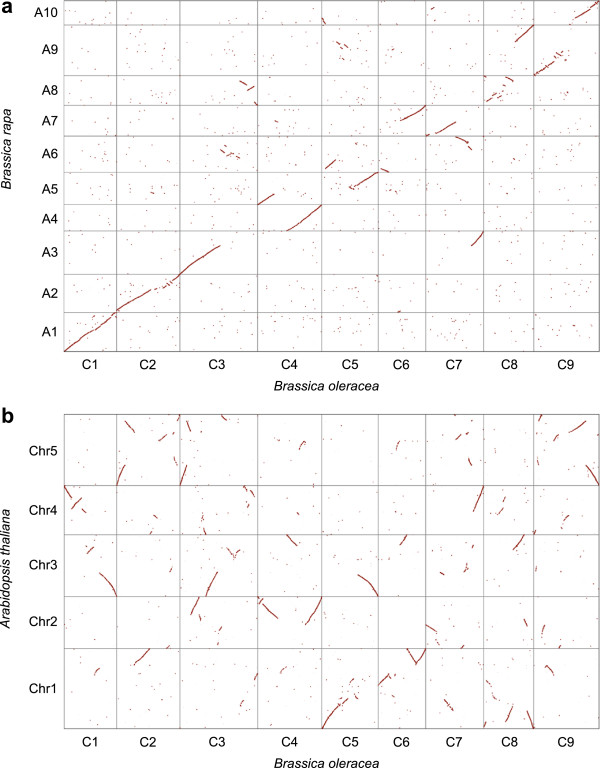
**Alignment of the *B. oleracea *genome with that of *B. rapa *and *A. thaliana*. (a)** Alignment with *B. rapa genome*; **(b)** alignment with *A. thaliana genome*. Dot-plots showing Nucmer alignments of stretches of sequence similarity between the genomes.

Alignment of the protein coding regions of *B. oleracea* with those of *B. rapa* provided markers to define the regions of conserved synteny between the two genomes. The conserved synteny closely followed the regions of homoeology previously identified in the amphidiploid *B. napus* (Figure 4a) [17]. In general a single *B. oleracea* pseudomolecule demonstrated strong conservation with large segments from not more than three *B. rapa* pseudomolecules, suggesting that a limited number (<20) of large scale rearrangements differentiate the two species. Chromosomes C8 and C9 showed more fractured alignment with A8 and A9, respectively; however, most of these localized rearrangements appear to be specific to the A genome since the same regions show conserved synteny between *B. oleracea* and *A. thaliana* and could represent errors in the earlier *B. rapa* assembly (Figure 4b).

**Figure 5:**
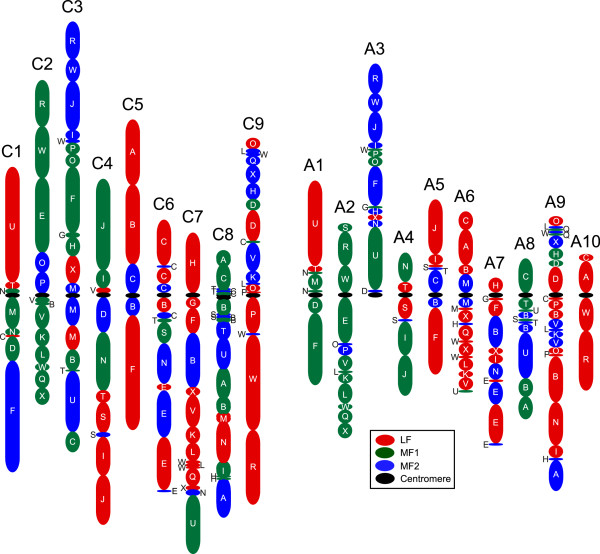
**Derived ancestral block structure for *B. oleracea *** **and *B. rapa*.**

Comparisons with the model *A. thaliana* identified the mesopolyploid event common to the *Brassica* species, with segments of the *A. thaliana* genome, representing the ancestral Brassicaceae blocks (A to X) [18,19], each being found in three copies in the *B. oleracea* genome (Figure 5; Table S7 in Additional file 2). Almost 90% (428.9 Mb) of the assembled and anchored genome was organized in ancestral blocks, with the remainder largely being restricted to pericentromeric regions and telomeres, where a lower gene density was found. Similar to observations for *B. rapa,* one copy (sub-genome) of each block had undergone less fractionation (or gene loss) than the remaining two (Table S7 in Additional file 2). The least fractionated (LF) sub-genome maintained 49.5% of the annotated *A. thaliana* gene copies in the equivalent blocks, while the other two sub-genomes maintained 35.3% (most fractionated - MF1) and 29.8% (MF2) of the gene content, respectively. The number of synonymous substitutions per synonymous site (*Ks*), which are thought to be immune from selective pressure, can approximate the natural mutation rate and by extrapolation the differential age of genomes. The *Ks* distribution for intra-subgenome pairs (LF, MF1 and MF2) and orthologous pairs with *A. thaliana* could not distinguish between the three genomes, and similar to previous reports suggested that these *Brassica* genomes had diverged from a common ancestor 23 Mya and from *A. thaliana* 27 Mya (Figure S3 in Additional file 1). The diploid *Brassica* genomes contain remnants of three older duplication events (α, β, γ), with the oldest γ gene copies being maintained in greater numbers than their more recent counterparts. This suggests selective pressure to retain the remaining copies, which is reflected in significantly lower *Ka*/*Ks* values for these genes (Figure S4 in Additional file 1).

### Single base resolution of the *B. oleracea* methylome

Sequence data from bisulfite-treated DNA extracted from TO1000 leaf tissue was analyzed to provide a picture of the cytosine DNA methylation landscape across the genome (Figure 3). From the almost 69 million trimmed sequence reads, 47.7% were uniquely mapped to the *B. oleracea* genome, providing an average read depth of 7.29 (per strand) at 93.5% of the cytosines in the assembled genome. Methylated cytosine bases were determined by applying a binomial test to data extracted from reads at each cytosine, using unmethylated lambda DNA as a control.

Cytosine methylation can be divided into three types based on sequence context, where mCG and mCHG (where H is A, C or T) possess symmetry and mCHH is asymmetric. The level of DNA methylation observed throughout the *B. oleracea* genome was 54.9% of all CGs, 9.4% of all CHGs and 2.4% of all CHHs. Varying reports of such levels have been found for plants ranging, for mCG, from 22% in *A. thaliana*[20,21], to 51% in soybean [22], and 59% in rice [20]. The *B. oleracea* genome was enriched for mCG when compared to levels more commonly observed for *A. thaliana* but demonstrated similar levels of cytosine methylation in the mCHG and mCHH contexts. The pattern observed in *B. oleracea* was in contrast to that noted recently for the paleopolyploid soybean, where higher levels of both mCG and mCHG were reported [22]. The distribution of methylated cytosines across the genome mirrored the distribution of repetitive elements, with methylation enrichment observed in particular towards the centromeric regions (Figure 3). This biased distribution is a reflection of the higher levels of cytosine methylation found in all contexts at the individual repetitive element level (Table S8 in Additional file 2). The average level of mCG methylation for repeat elements ranged from 88% to 100%, with SINEs showing the highest levels. mCHG and mCHH are commonly associated with silencing of repetitive elements, and although showing more variation compared to mCG, they were consistently two to seven times the observed genome-wide levels across all repeat types (Table S8 in Additional file 2).

**Figure 6:**
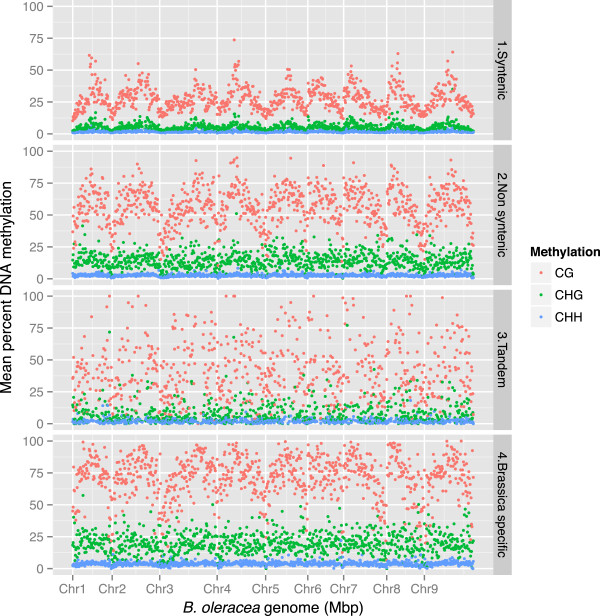
**Cytosine methylation levels across specific categories of genes of the *B. oleracea *genome.** The mCG (red), mCHG (green) and mCHH (blue) levels are shown for each gene model (includes promoter regions, UTRs, exons, introns and 3′ flanking), based on a sliding window of 500 kb.

The average cytosine methylation varied across gene structures, with higher levels observed upstream and downstream of the predicted translational start and stop sites (Figure S5 in Additional file 1). Interestingly, and in contrast to *A. thaliana*[20], higher levels of cytosine methylation, in particular mCG, were observed for intronic sequences compared to exons (Table S8 in Additional file 2). However, the most marked differences were observed between syntenic and non-syntenic predicted genes. A total of 16,601 non-syntenic genes were identified, which were either outside of ancestral blocks or were within block boundaries but lacked a syntenic orthologue in *A. thaliana* and were homologous to an *A. thaliana* gene from elsewhere in the genome. Non-syntenic genes could result from transposition of single genes or small segmental translocations of multiple linked genes. Cytosine methylation in all contexts was significantly enhanced across non-syntenic genes (Figure 6; Figure S6 in Additional file 1). Additional evidence, including generally high *Ka*/*Ks* levels (Figure 3), indicating low purifying selection, shorter average gene length and lower average number of exons, suggests that the non-syntenic genes may be undergoing pseudogenization (Figure S7 in Additional file 1). Similar observations were made for genes repeated in tandem and for *B. oleracea*-specific genes (Figure 6; Figure S6 in Additional file 1).

### Correlation of cytosine methylation and expression levels

**Figure 7:**
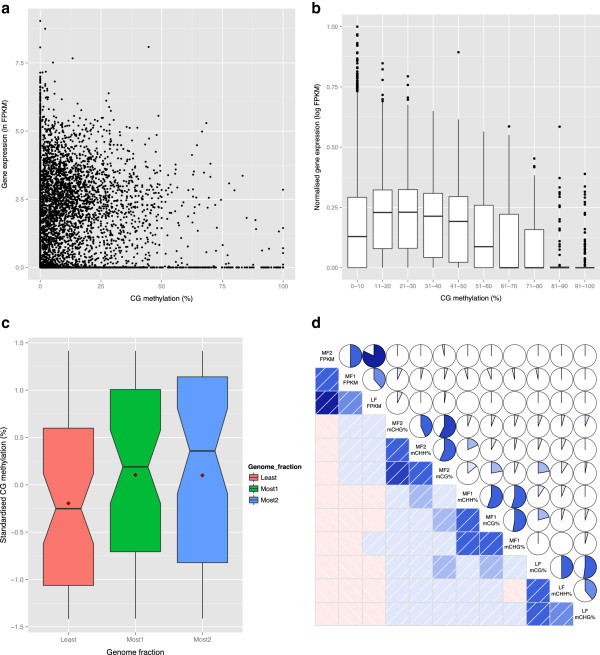
**Correlation of methylation status with gene expression and genome triplication in *B. oleracea*. (a)** Expression levels (log(FPKM)) plotted against mCG gene body methylation levels. **(b)** Box plot representation of different levels of mCG gene body methylation in syntenic genes (along x-axis) with normalized gene expression levels plotted on the y-axis. **(c)** Box plot representation of different levels of mCG observed across the three sub-genomes. **(d)** Correlation of gene expression (FPKM) and methylation levels among the fully retained orthologues of the three genomes. Below the diagonal, positive and negative pair-wise correlations are indicated in blue and red, respectively. Darker coloring indicates a greater magnitude for the correlation. Above the diagonal, the color and extent of the filled area of each of the pie-charts represents the strength of each pair-wise correlation. Positive and negative correlations are indicated by the pie being filled in a clockwise or anticlockwise direction, respectively.

Expression levels assessed using FPKM (fragments per kilobase of exon per million fragments mapped) values calculated from leaf transcriptome RNASeq data showed a non-linear relationship to mCG levels across the gene body (exons and introns) (Figure 7a). Although high expression tends to be associated with lower methylation levels, there were exceptions, with non-expressed genes showing limited methylation and heavily methylated genes being expressed, but in general moderately expressed genes showed some level of gene body methylation. Gene body methylation (mCG specifically) of expressed genes has been shown to be conserved across numerous species yet its role has yet to be determined [20]. In *B. oleracea* moderate CG gene body methylation was similarly associated with higher gene expression (Figure 7b; Figure S8 in Additional file 1).

### Impact of the Brassiceae-specific hexaploidy event

The most recent WGD event(s) in related *B. rapa* resulted in a hexaploid structure composed of three sub-genomes differentiated not only by extensive chromosomal restructuring but also varying levels of gene fractionation (or loss). This WGD preceded the divergence of *B. oleracea* from *B. rapa* and except for a limited number of additional gross chromosomal rearrangements both species have maintained a similar genome structure. In addition, comparable levels of biased gene fractionation have resulted in one sub-genome maintaining higher numbers of orthologous genes relative to the other two. It has been suggested that genome dominance post-polyploidy determines the fate of duplicated genes and gene expression has been positively correlated with maintenance of gene copies in other species and may play a similar role in *Brassica* species [23].

**Figure 8:**
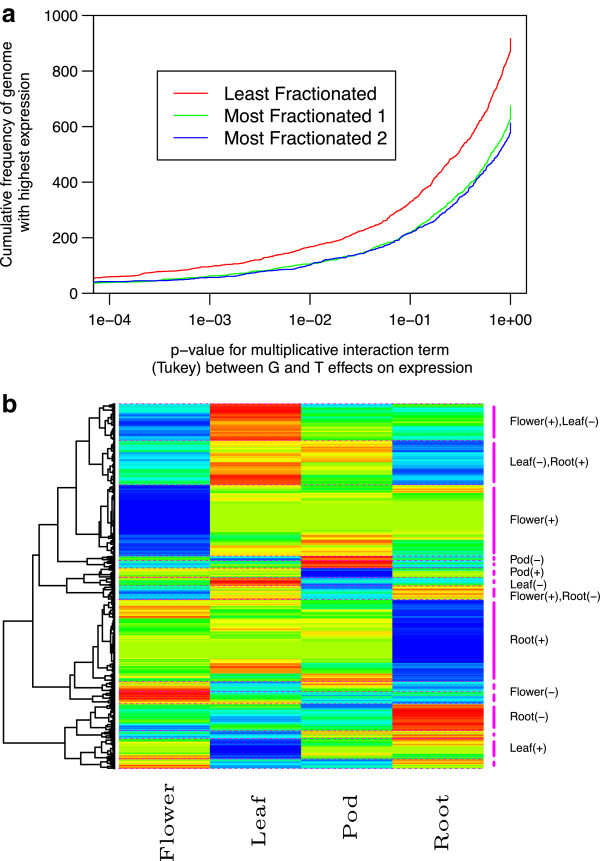
**Genome dominance and functional diversification of *B. oleracea *homologues retained across three sub-genomes. (a)** Cumulative frequency of homologous genes belonging to the three sub-genomes with highest expression across all tissue types. *P*-values were calculated for interaction between sub-genomes (G) and tissue-type (T) effects on expression. **(b)** Hierarchical clustering of gene expression profiles for fully retained triplicated genes across four tissue types. Red and blue indicate lowest and highest expression values, respectively. Intermediate expression values follow a rainbow coloring pattern. The dotted lines to the right correspond to partitioning of the genes into 15 clusters.

After fractionation only 2,242 genes have been fully retained across the three sub-genomes of *B. oleracea*. This subset of genes was used to detect any residual effects of genome dominance on gene expression (Figure 8a). Analysis of sub-genome (G) by tissue-type (T) interactions across four tissue types identified 26% (588) of the fully retained triplets with a statistically significant effect (*P* < 0.05; Tukey test for interaction). Of these 588 triplets, 43% showed higher expression in the LF sub-genome, while 29% and 28% showed higher expression in MF1 and MF2, respectively, indicating an expression bias for the LF. Hierarchical clustering of expression patterns for 5,872 fully retained genes showing expression variation across all four tissues resolved 15 clusters, which showed tissue-specific patterns (Figure 8b). The majority of the triplicated genes (83%) were separated across clusters signifying independent regulation of gene copies, which implies post-polyploidy mechanisms such as neo- or sub-functionalization are acting on the gene duplicates.Since cytosine methylation appears to play a role in regulating gene expression, levels of cytosine methylation were determined at both the sub-genome level and across the fully retained triplicated gene copies. At the sub-genome level there was no significant difference in gene density, yet assessing methylation across ancestral blocks indicated that although not significantly different, the levels of methylation in all contexts were lowest for the least fractionated genome (Figure 7c). However, there was no correlation or bias observed between cytosine methylation in any context across the gene body with sub-genome copy (Figure 7d). The bias in methylation observed at the sub-genome level could reflect an earlier trend towards suppression of the more fractionated genomes and the process of gene fractionation may have removed the requirement for continued involvement of cytosine methylation in maintenance of gene expression levels for the remaining duplicated copies. Although no evidence of genome dominance was observed at the gene level, each sub-genome copy appeared to have independent patterns of cytosine methylation, which could provide further evidence of functional diversification (Figure 7d). The order of pairwise correlations in Figure 7d was determined using principle component analysis, which clustered similar patterns, showing greater correlation between the context of cytosine methylation within the sub-genomes than between methylation patterns across the sub-genomes.

## Discussion

The *B. oleracea* species include a wide range of important vegetable crops, with diverse morphological variation and an eclectic mix of phytochemicals and secondary metabolites, many with health promoting properties. Of these morphotypes, TO1000, a kale-like plant, was selected as an excellent experimental model since it is rapid cycling, self-compatible, uncommon within *B. oleracea*, and has associated genomic and genetic resources, including a BAC-based physical map [24], a doubled haploid mapping population [25] and a mutagenized population [26]. Thus, the reference genome sequence of TO1000 will provide an excellent tool for dissecting the molecular basis of the remarkable variation found within the species. In addition, *B. oleracea* is a close relative of the C genome species that hybridized with *B. rapa* to form the economically important oilseed crop *B. napus*; as such, the genome sequence will provide a foundation for further research in the allopolyploid relative.

The assembled nuclear genome sequence of TO1000 indicates that the increased genome size of *B. oleracea* compared to *B. rapa* results from multiplication of both repetitive and genic sequences. However, the two genomes evolved from a common ancestor and, as such, are remarkably similar with minimal large-scale chromosomal rearrangements having occurred since their divergence approximately 4 Mya [2]. The two genomes share the Brassiceae-specific hexaploidization event and 77.8% of the annotated genes from *B. rapa* have a retained *B. oleracea* orthologue. Thus, even with on-going independent fractionation post-polyploidization and species divergence there is significant conservation of syntenic gene content between the A and C genomes, which is reflected in the composition of ancestral blocks along with their fractionation status in the two genomes (Figure 5). The maintenance of the syntenic block structure reflects the recent shared ancestry of the two genomes. The mechanism by which the non-syntenic genes have evolved is unclear, although they now show evidence of pseudogenization. It has been suggested that such changes could result from transposon activity causing transduplication [27], and there was a slight bias in prevalence of transposable elements flanking non-syntenic genes; however, 30% of syntenic genes also had transposable elements annotated within 500 bp of the coding region, which largely reflects the higher density of such structures in the *B. oleracea* genome (Table S9 in Additional file 2). It appears that the majority of the gene loss observed in the two *Brassica* diploids occurred prior to species divergence. Although the chromosomal rearrangements and independent repetitive element evolution may be sufficient to ensure inter-specific incompatibility, the majority of the species morphological characteristics presumably stem from allelic diversification and expansion of particular gene families.

Polyploidy is a prevalent evolutionary mechanism and it has been suggested that most angiosperms have experienced one or more polyploidy events in their history [28]. *Brassica* species are excellent models for the study of this mode of genome evolution since at least four WGD events underlie their current genome architecture, three shared with the Brassicaceae experimental model *A. thaliana* and the most recent (estimated at approximately 23 Mya) specific to Brassiceae. The genome sequence of *B. rapa* allowed a detailed analysis of the three ancestral genomes that had fused to form the Brassiceae tribe, revealing an asymmetrical pattern of gene loss, suggesting a two-step polyploidy event with the last genome to join retaining the highest number of ancestral genes [6]. The genome of *B. oleracea* mirrors this observation. It has been proposed that the genome dominance inferred to regulate this biased gene loss stems from imbalances in gene expression [23] and our analyses of transcriptome data from four *B. oleracea* tissue types supports this hypothesis, since higher expression of genes from the LF genome was observed for 43% of the fully retained triplets showing a significant sub-genome by tissue type interaction. However, study of the retained triplicated genes provides more compelling evidence for functional diversification of gene duplicates, with 83% of the triplicated genes showing independent expression patterns across the four tissue types. The fate of duplicate gene copies following WGD has been the subject of much debate and numerous models have been proposed to explain their retention or loss with discussion seemingly favoring dosage balance, neo-functionalization and sub-functionalization [29,30]. Perhaps at one time controlled through dosage balance, which would require coordinated expression, the maintenance of duplicate genes post-hexaploidy in *B. oleracea* appears now to result from neo- or sub-functionalization.

The mechanisms that respond to WGD to provide meiotic stability post-polyploidy are unknown but the rapidity of change offered by epigenetic effects has suggested their potential role [31]. Indeed, methylation re-patterning post-hybridization has been found in specific plant lineages, including *Brassica* species [32]. Cytosine methylation has been shown to have an impact on gene expression and its role in controlling the activity of transposable elements could influence genome stability. In addition, cytosine methylation itself can be a source of further molecular variation through spontaneous deamination of methylated cytosines. Through single base resolution we have provided the first cytosine methylation map for the reference *B. oleracea* genome, which parallels findings in other eukaryotic genomes. Biased distribution reflecting the role of cytosine methylation in repeat element suppression was observed and patterns of methylation across gene structures were similar to those observed for multiple species [20]. Although methylation across the three ancestral sub-genomes was imbalanced, with the least fractionated genome experiencing lower levels, this did not appear to be reflected at the level of the retained triplicate genes. Since the triplicate genes appear to have undergone a significant level of functional diversification, the influences of residual genome dominance from cytosine methylation seem minimal at the gene level. If cytosine methylation was an influencing factor in restoring balance in the nucleus of the ancestral polyploid *B. oleracea*, its effects are no longer apparent. It has been shown that methylation patterns can be conserved across orthologous genes within lineages [33] yet the patterns observed for the fully retained triplicated homologues in *B. oleracea* appeared largely independent. Thus, it is possible that cytosine methylation played a significant role early in the ancestry of *B. oleracea* to quickly establish relative expression differences but may now be involved in diversification of gene expression among the three sub-genomes.

## Conclusions

We present a reference genome for the mesopolyploid *B. oleracea*, a resource for further study of polyploid evolution and a platform for genetic improvement of an array of Brassica vegetable crop types. Associated transcriptome data suggest the functional diversification of duplicate gene copies within the genome could be a source of the rich diversity within this species. In addition, the cytosine methylation landscape for this mesopolyploid provides insights into the role of this genome-wide mechanism in the evolution of the *B. oleracea* genome. Our findings show empirical links between gene fractionation, expression and epigenetic phenomena as major factors shaping the evolution of a polyploid genome.

## Materials and methods

### Library construction and sequencing

A homozygous doubled haploid line TO1000DH3 derived from *B. oleracea* cultivar TO1434 was chosen for sequencing. High quality nuclear DNA was extracted from young leaves using a megabase-sized DNA isolation protocol as described in [34]. Briefly, approximately 40 g of fresh leaf tissue was homogenized in 200 ml buffer (HB; 0.01 M Trizma base, 0.08 M KCL, 0.01 M EDTA, 1 mM spermidine, 1 mM spermine, 0.5 M sucrose plus 0.15% β-mercaptoethanol, pH 9.4 to 9.5). The homogenate was filtered and the nuclei pelleted by centrifugation (1,800 g at 4°C for 20 minutes). The pellet was resuspensed (1 × HB plus 0.5% Triton-×100) and centrifuged three times. Finally, the nuclei were resuspended in 10 ml lysis buffer (100 mM TrisCl, 100 mM NaCl, 50 mM EDTA, 2% SDS). High molecular weight genomic DNA was extracted by traditional proteinase K (0.05 mg/ml; 65°C for 2 h) digestion followed by RNAase A treatment, two cycles of phenol/chloroform extraction and ethanol precipitation. Quantification of genomic DNA was performed using PicoGreen dsDNA kit (Molecular Probes, Life Technologies Inc., Burlington, ON, Canada). Genomic DNA (5 to 40 μg) was randomly sheared using one of: Covaris S2 ultrasonicator (Covaris Inc., Woburn, MA, US); Hydroshear (Genomic Solutions Inc., Ann Arbor, MI, US); or gas-driven nebulizers. For Illumina sequencing, four paired-end (PE) libraries (with median insert sizes of 273, 335, 418 and 532 bp; Table S1 in Additional file 2) and five short-span mate-paired (MP) libraries (from 2.5 to 8.5 kb) were constructed following the manufacturer’s instructions (TruSeq DNA sample preparation and MP library preparation kit v2 (Illumina, San Diego, CA, US), respectively). Libraries were size selected using the Pippin prep automated gel electrophoresis system (Sage Science, Beverly, MA, US), quantified using a BioAnalyzer (Agilent Technologies, Mississauga, ON, Canada) and KAPA library quantification kit for Illumina (KAPA Biosystems, Wilmington, MA, US), and sequenced from both ends (paired-end) for 100 cycles on an Illumina HiSeq 2000 instrument. For 454 pyrosequencing, two medium span MP libraries with median insert sizes of 8 and 17 kb (Table S1 in Additional file 2) were constructed following the method described in the GS FLX Titanium 20 kb span PE library preparation manual from Roche and sequenced using a Roche 454 FLX Titanium sequencer (454 Life Sciences, Branford, CT, US).

### Genome assembly

Initially, all Illumina and 454 reads were filtered for adapter contamination, PCR duplicates, ambiguous residues (N residues) and low quality regions. The initial backbone of the draft genome was assembled with Illumina reads using *De Bruijn* graph*-*based SOAPdenovo (version 1.05) assembler [8,35], run with a k-mer parameter of 47 and each library ranked according to insert size from smallest to largest. The gaps within assembled scaffolds were filled with the short insert PE reads using GapCloser (version 1.12). The resulting assembly consisted of a total of 35,436 contigs and short scaffolds, with a sequence span of 488 Mb and an N50 size of 265 kb. BAC end sequences for TO1434 were downloaded from NCBI (LIBGSS_011756) and trimmed for quality, ambiguous bases and adapter sequences. Bambus [9] was used to overlay all the 454 MP information and the BAC end sequence data onto SOAPdenovo scaffolds to improve scaffold lengths as described in [34]. In short, all 454 MP reads and BAC end sequence reads (Table S1 in Additional file 2) were aligned to the scaffolds using a genomic mapping and alignment program (GMAP) [36]. The output from GMAP was used to create a Bambus-compatible GDE formatted contig file that indicated scaffold links. Redundant or multi-mapped mates, mates where only one read mapped, and those where both mates mapped to a single scaffold were considered invalid links. Each link was considered in Bambus in ascending order of their length, with scaffolding parameters including a redundancy level of 3 and link size error of 5%. Any potentially ambiguous scaffolds were resolved using the 'untangle' utility of Bambus. Bambus was able to order, orient and merge 2,623 of these pre-assembled SOAPdenovo scaffolds into 646 superscaffolds, resulting in a greatly improved assembly with an N50 size of 850 kb.

### Construction of a high density genetic map and anchoring of the genome

A high density genetic map representing nine linkage groups was constructed using a mapping population of 94 doubled haploid lines (DH) derived previously from a cross between TO1000 and Early Big [25]. A total of 2,299 polymorphic loci (SNPs, simple sequence repeats and insertion/deletion polymorphisms) identified using the RAD approach [11] were used to integrate assembled scaffolds with the genetic map. Collinearity between *B. oleracea* provisional pseudomolecules and *A. thaliana* and/or *B. rapa* was used to further assist with ordering and orientation of scaffolds for which there was paucity of adequate genetic recombination and markers. A total of 66 instances of false joins or insertions within Bambus superscaffolds were identified based on marker discontiguity and collinearity information. These scaffolds were split and the correct position of each of the fragments was determined based on marker and collinearity information. Final scaffolds were renamed as ‘Scaffold’ and numbered sequentially based on their length from longest to shortest. The order and orientation of scaffolds within each pseudomolecule were determined based on marker order within each scaffold, and marker contiguity pattern between adjoining scaffolds. Scaffolds with too few markers were ordered and oriented using collinearity information. The final version of the draft genome representing nine pseudochromosomes and 32,919 unanchored scaffolds was collated using a custom Perl script (Additional file 3), and the ordering and orientation information of scaffolds within each pseudochromosome was compiled in AGP files. The quality of the assembled genome was ascertained by performing several independent tests (Table S3 in Additional file 2; Figure S2 in Additional file 1).

### Half-tetrad genetic mapping of centromeres

Centromere positions were mapped using half-tetrad analysis in a population of 49 plants derived from first-division restitution unreduced gametes of several *B. napus* × *B. carinata* hybrids [37]. A total of 13,098 SNPs polymorphic between the *B. napus* and *B. carinata* parent genotypes for at least two progeny sets were selected from the recently released Illumina *Brassica* 60 K SNP chip and used to assess offspring heterozygosity at each C-genome locus. Increasing heterozygosity towards each centromere was used to predict centromere locations (see [38] for details).

### Transcriptome sequencing

The whole plant transcriptome of *B. oleracea* based on Illumina RNAseq data was characterized for four different tissue samples (leaf, root, flower, and pod; Table S4 in Additional file 2). Total RNA was isolated using the RNeasy plant mini kit (QIAGEN, Toronto, ON, Canada), including on-column DNase digestion, according to the manufacturer’s instructions. The integrity and quantity of total RNA was assessed using RNA 6000 Nano labchip on the BioAnalyzer (Agilent). Sequencing libraries were constructed following the standard TruSeq RNA sample preparation guide (Illumina), and PE sequencing was performed using the Illumina Hiseq 2000 platform. A total of 12.5 Gb raw RNAseq data were generated. Prior to read mapping, all reads were filtered for adapter contamination, ambiguous residues (N residues) and low quality regions. *B. oleracea* gene expression was assessed using a previously described TopHat and Cufflinks-based method [39]. For both TopHat and Cufflink analysis, maximum and minimum intron lengths were set at 2,500 and 20 bp, respectively. Transcript abundance was measured as FPKM values.

### Gene annotation

For accurate annotation of gene models, an integrated computational approach based on two major genome annotation pipelines, Maker [13] and PASA [40], was adopted. Maker provides a simplified process for aligning expressed sequence tags (ESTs) and proteins to the genome, and integrates this external homology evidence with *ab initio* gene predictions to produce polished gene annotations with evidence-based quality statistics. Inputs for Maker included the repeat-masked *B. oleracea* genome assembly (masked against ‘te_proteins.fasta’ in the Maker package, which contains a generic list of common transposable elements (TEs)), PlantGDB ESTs from *B. oleracea*, *B. rapa* and *B. napus*, and Uniprot (SwissProt + TrEMBL) plant protein database. *Ab initio* gene predictions were made by Fgenesh [40] and Augustus [41]. Maker gene structure annotations were further updated by PASA using evidence from Sanger ESTs and multiple *de novo* RNA-seq assemblies. Post-Maker processing included splitting potentially fused genes, extending genes, resolving internal rearrangements, trimming overlapping genes, and removing proteins of less than 50 amino acids and with no BLAST match to *A. thaliana*. The final annotation set contained a total of 59,225 gene models. Protein names are assigned using AHRD pipeline [42] with names extracted from BLASTP hits in TAIR v10, Swissprot and TrEMBL databases (queried in March 2012).

### Repeat annotation

A TE database constituted from *de novo* analysis of *B. napus* (Chalhoub *et al*., in preparation) was merged with databases of TEs previously constructed from analysis of *B. rapa*[1] and *B. oleracea*[43]*.* The TEs were classified into major subclasses and superfamilies (Table 3) based on their structural features. Inside each superfamily, elements sharing more than 80% sequence identity over more than 80% of their length, and at least 80 bp, are considered as belonging to the same family [44]. The merged TE database was used for comparative repeat masking [45] of the *B. oleracea* and *B. rapa* genomes.

### Synteny analysis

Sequence homology was detected by BLASTP of the predicted proteins against the *A. thaliana* proteome. BLAST hits with E-value 1E-20 or better and within the top 40% from the best bit score were kept for further analysis. The chains of syntenic *B. oleracea*-*A. thaliana* gene pairs were computed by DAGChainer [46] using the default parameters. In the case of a *B. oleracea* gene participating in more than one syntenic chain due to duplication in the *A. thaliana* genome, the *B. oleracea*-*A. thaliana* pair in the weaker scoring chain was removed from the analysis. The syntelog table was generated by placing the syntenic chains onto the *B. oleracea* chromosomes.

### Analysis of expression divergence

All calculations were completed using R [47]. For the 2,242 triplets of (fully retained) *B. oleracea* genes the raw expression values (FPKM) from each of the four tissue types (T) and three sub-genomes (G) were transformed by adding 1 and taking the natural logarithm. All subsequent calculations were done with the transformed values. The means for each sub-genome were taken over the four tissue types, and the genome with the highest expression was recorded. With no replication interaction (G×T) cannot be separated from random error, so the gene triplets were sorted by the *P*-value for an extra interaction term (Tukey) [48] that is the product of the main effects terms in the ANOVA model for the expression data for a gene triplet. The cumulative frequency of each genome with the highest expression was plotted against the Tukey *P*-values.

Hierarchical clustering of gene expression was carried out using 1 minus the Pearson sample correlation coefficient as the distance measure, which requires the variances of the expression values for each gene to be non-zero. This clusters correlated sets of expression values regardless of the absolute magnitude of the expression levels. The average linkage method was used to form the clusters.

### Protein family classification

An all-against-all BLASTP (E-value cutoff of 1E-10) [49] similarity search was performed among predicted protein sequences of four completely sequenced Brassicaceae species, *A. thaliana*, *A. lyrata*, *B. rapa* and *B. oleracea*. The pairwise similarities generated by this analysis were parsed and stored in a matrix that served as input for clustering by the TRIBE-MCL approach [50]. The groups of homologous proteins were clustered using Markov cluster (MCL) algorithm at an inflation rate (I) of 3.0 and other default parameters. A χ^2^ test was performed to determine if significant expansion of gene families among the Brassicaceae species occurred. χ2 probability values were adjusted by Bonferroni correction and the post-hoc test was conducted only if the *P*-value after Bonferroni correction was <0.05.

### Single base resolution methylome sequencing

Genomic DNA was isolated from *B. oleracea* TO1000 nuclei prepared according to the method found in Zhang *et al*. [51] from leaf tissue as above. TO1000 genomic DNA (5 μg), along with 0.5% w/w of unmethylated lambda DNA (Promega, Madison, WI, US) included to assess bisulfite conversion efficiency, was sheared using the Diagenode Bioruptor Plus to produce an average fragment size of 300 bp. Library construction was performed using the Illumina Paired-End Sample Prep Kit and QIAGEN PCR Purification kits according to the Illumina Whole-genome Bisulfite Sequencing for Methylation Analysis protocol. Methylated adapters from the Illumina TruSeq DNA Sample Prep Kit v2 were substituted for the unmethylated adapters within the Illumina Paired-End Sample Prep Kit. Ligation products equivalent to 450 bp were purified using the BluePippin DNA size selection system (Sage Science). Bisulfite conversion of this DNA was performed using the QIAGEN EpiTect Bisulfite kit. The libraries were quantified using a BioAnalyzer (Agilent) and KAPA library quantification kit for Illumina (KAPA Biosystems), and sequenced from both ends (paired-end) for 100 cycles on either an Illumina HiSeq 2000 or Illumina HiScanSQ.

BSMAP (v2.74) and an associated python script (methratio.py) [52] were used to analyze cytosine methylation ratios. All reads were trimmed for quality and adapter sequence, potential PCR duplicates were removed and only those reads mapping uniquely to the genome were retained. Sequencing of cytosines in lambda DNA included in each library provided an error rate for non-conversion; this rate was used in a custom Perl script to identify methylated cytosines using the binomial probability distribution at a false discovery rate of <5% (Additional file 4).

Perl was used for text manipulation to assign pseudochromosome coordinates to gene features, 5′ promoter, 5′ UTR, gene, exon, intron, 3′ UTR and 3′ regions, and incorporate these annotations in a custom .gff file. A combination of Perl and R was used to perform statistical analyses on these data. Graphics summarizing the observed statistical relationships were developed using ggplot2, RGL and corrgram in R [47]. All relevant Perl and R scripts are open source and freely available upon request.

### Accession numbers

All the raw data used in the assembly of the genome, the RNASeq data used in the expression analysis, and the bisulfite sequence data have been deposited at DDBJ/EMBL/GenBank short read archives under accession numbers SRP040796, SRS586190, SRS586272, SRS586273. The whole genome shotgun project has been deposited at DDBJ/EMBL/GenBank under the accession JJMF00000000. The version described in this paper is version JJMF01000000.

## Abbreviations

BAC: bacterial artificial chromosome; bp: base pairs; EST: expressed sequence tag; FPKM: fragments per kilobase of exon per million fragments mapped; GBS: genotyping-by-sequencing; LF: least fractionated; MF: most fractionated; MP: mate-paired; Mya: million years ago; PE: paired-end; RAD: restriction site-associated DNA; SINE: short interspersed nuclear element; SNP: single nucleotide polymorphism; TE: transposable element; UTR: untranslated region; WGD: whole genome duplication.

## Competing interests

The authors declare that they have no competing interests.

## Authors’ contributions

CK and HT completed the genome assembly and annotation. VK and SLB assisted with the genome annotation. MGL assisted with the genome assembly. SK, WEC and JN carried out bioinformatic analyses. TB generated methylome data and SJR analysed these data, assisted by WEC. BC led and coordinated the repeat element annotation completed by FD, HB and JJ. CT, BC, JCP, IB, GK and GB participated in the design of the study and contributed sequence data. CC and TH generated materials and data. ASM and MNN assisted with mapping of the centromeres. XW and AHP physically mapped the genome. JM, PGW, SM, JB and DE assisted with validating the genome. IAPP and AGS conceived of the study, participated in its design and coordination and drafted the manuscript. All authors read and approved the final manuscript.

## Supplementary Material

Additional file 1: Figure S1 estimation of genome size based on analysis of 17 bp k‒mer frequency. **Figure S2.** dotplots representing nucmer alignments of regions of sequence similarity between 18 previously sequenced *B. oleracea* BACs and the *B. oleracea* genome sequence. **Figure S3.***Ks* distribution for orthologous gene pairs between **(a)***A. thaliana* and each of the *B. oleracea* sub‒genomes; and **(b)** between each of the *B. oleracea* sub-genomes. **Figure S4.** percentage retention of WGD duplicate gene copies in the *B. rapa* (Br) and *B. oleracea* (Bo) genomes. **Figure S5.** cumulative cytosine methylation levels across annotated *B. oleracea* genes. **Figure S6.** cytosine methylation levels across specific categories of genes of the *B. oleracea* genome. **Figure S7. (a)** average gene length, **(b)** exon number and **(c)** intron number for syntenic, non‒syntenic, tandem and *B. oleracea*-specific genes. **Figure S8.** box plot representation of different levels of **(a)** mCHG and **(b)** mCHH gene body methylation in syntenic genes (normalized gene expression levels). Click here for file

Additional file 2: Table S1 TO1000DH3 sequencing library statistics. **Table S2.** assessment of completeness, continuity and coverage of gene space in *B. oleracea* assembly by CEGMA analysis. **Table S3.** distribution of orthologues (best-blast hit candidates) of *B. oleracea* genes in other plant genomes. **Table S4.** expression analyses of *B. oleracea* based on RNASeq data. **Table S5.** analysis of expansion of gene families in the Brassicaceae species. **Table S6.** putative centromere locations in *B. oleracea* chromosomes. **Table S7.** syntenic ancestral block structure between *A. thaliana* and three sub-genomes of *B. oleracea.***Table S8.** level of methylated cytosines over the cumulative regions of **(a)** repetitive elements, **(b)** all annotated genes, **(c)** syntenic genes, **(d)** non-syntenic genes, **(e)** tandemly repeated genes and **(f)***B. oleracea* specific genes. **Table S9.** number of transposable elements (TEs) found within **(a)** 1 kb and **(b)** 500 bp 5′ and/or 3′ of annotated *B. oleracea* genes. Click here for file

Additional file 3 Perl script for generation of pseudochromosomes. Click here for file

Additional file 4 Perl script to identify methylated cytosines using the binomial probability distribution. Click here for file
