# Supplementary material for: Transcriptome and methylome profiling reveals relics of genome dominance in the mesopolyploid Brassica oleracea
Source: Genome Biol. 2014 Jun 10;15(6):R77. doi: 10.1186/gb-2014-15-6-r77 (PMC4097860; doi:10.1186/gb-2014-15-6-r77)

Figure S1: Estimation of genome size based on analysis of 17 bp k-mer frequency[1].

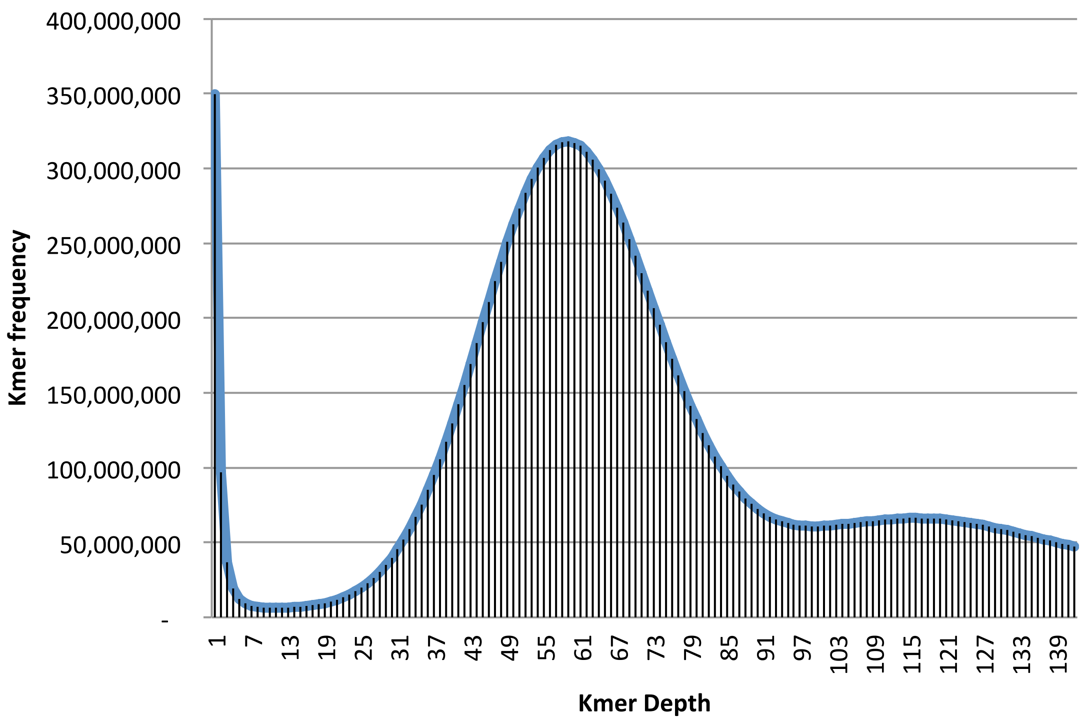

1. Analysis of k-mer frequency distribution in short read data can provide an estimate of the sequencing depth that then can be utilized to determine the genome size. The real sequencing depth (N) is correlated with the peak of the k-mer frequency (M), read length (L) and k-mer length (K), and can be derived using the formula N=M * L/(L−K+1). To determine the genome size of *B. oleracea* TO1000, the occurrences of 17 bp k-mers in 48.6 Gb of filtered Illumina PE data were counted using Jellyfish (Marcais and Kingsford, 2011) . Genome size was estimated by dividing the total length of sequencing reads used in the analysis by sequencing depth. This analysis suggested a genome of 648 Mb, which is in agreement with the range of flow cytometry based estimates, from 599 to 696 Mb (http://www.brassica.info/info/reference/genome-sizes.php).

Marçais G, Kingsford C. (2011) A fast, lock-free approach for efficient parallel counting of occurrences of k-mers. Bioinformatics. 27(6):764-70.

Figure S2: Dot-plots representing nucmer alignments of regions of sequence similarity between 18 previously sequenced *B. oleracea* BACs (y-axis) and the *B. oleracea* genome sequence (x-axis)

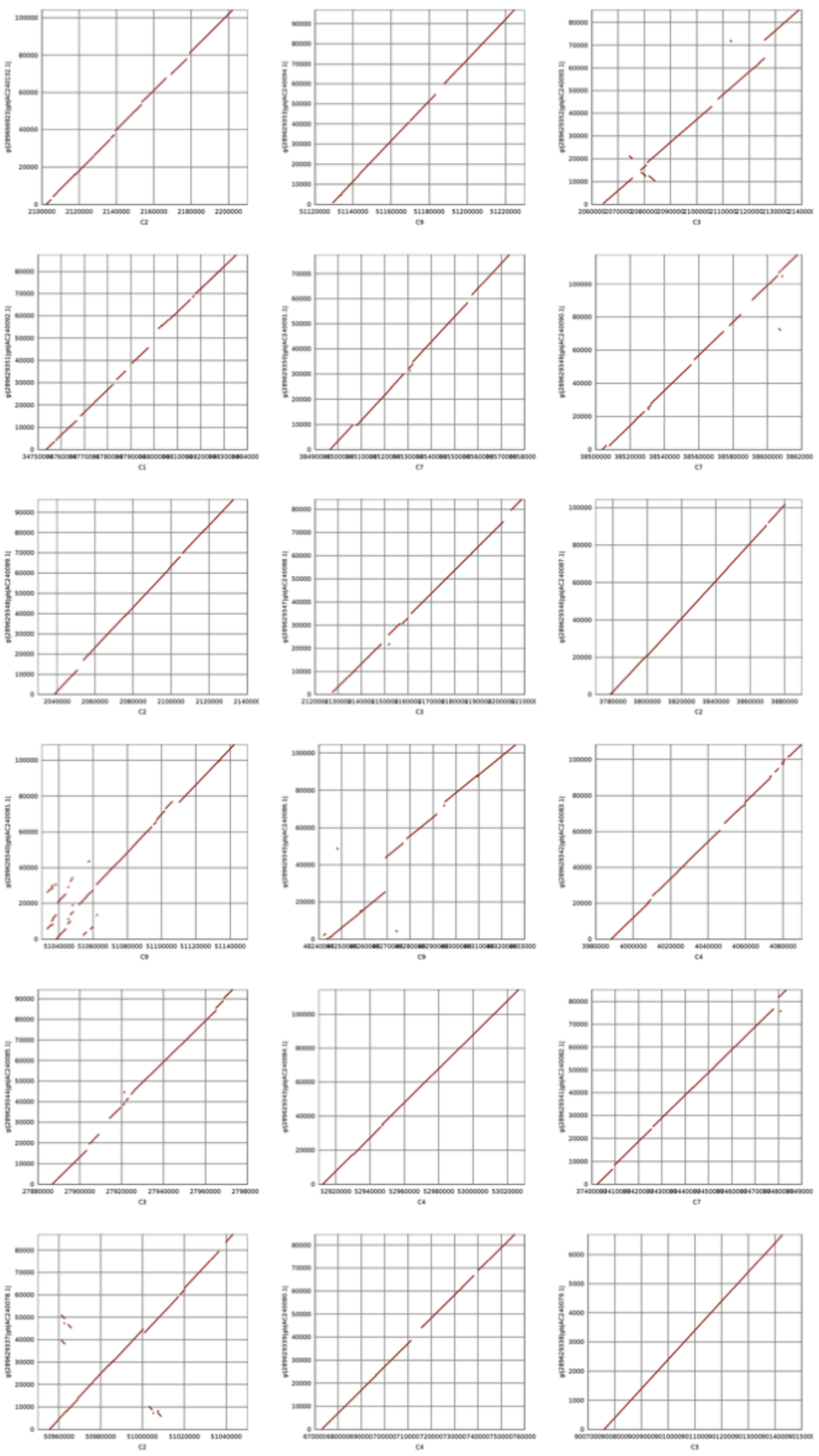

Figure S3: Ks distribution for orthologous gene pairs between: a) *A. thaliana* and each of the *B. oleracea* sub-genomes; and b) between each of the *B. oleracea* sub-genomes.

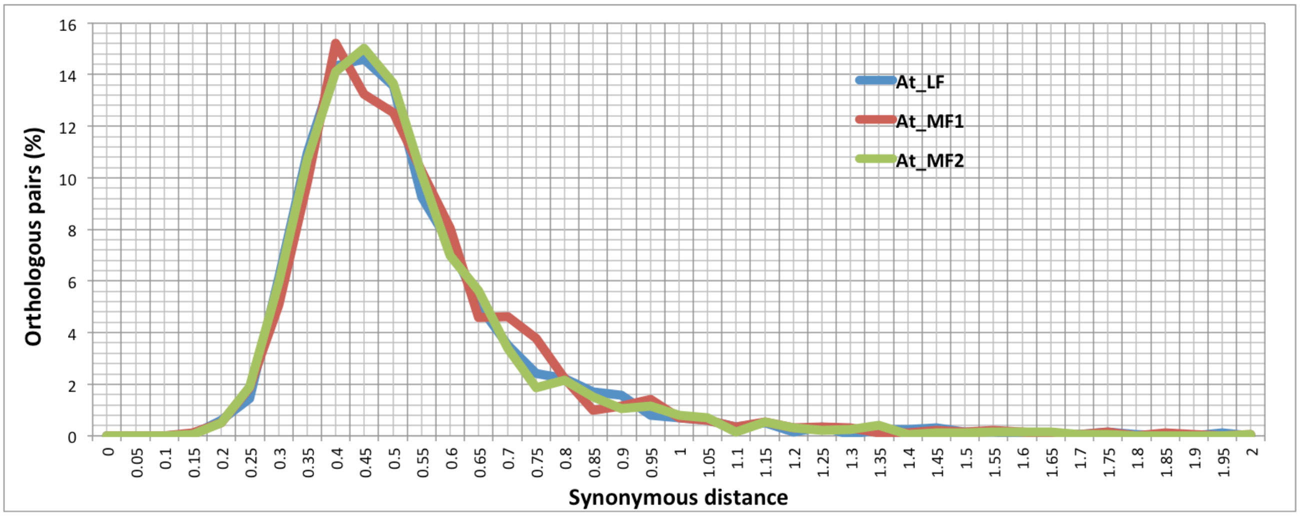

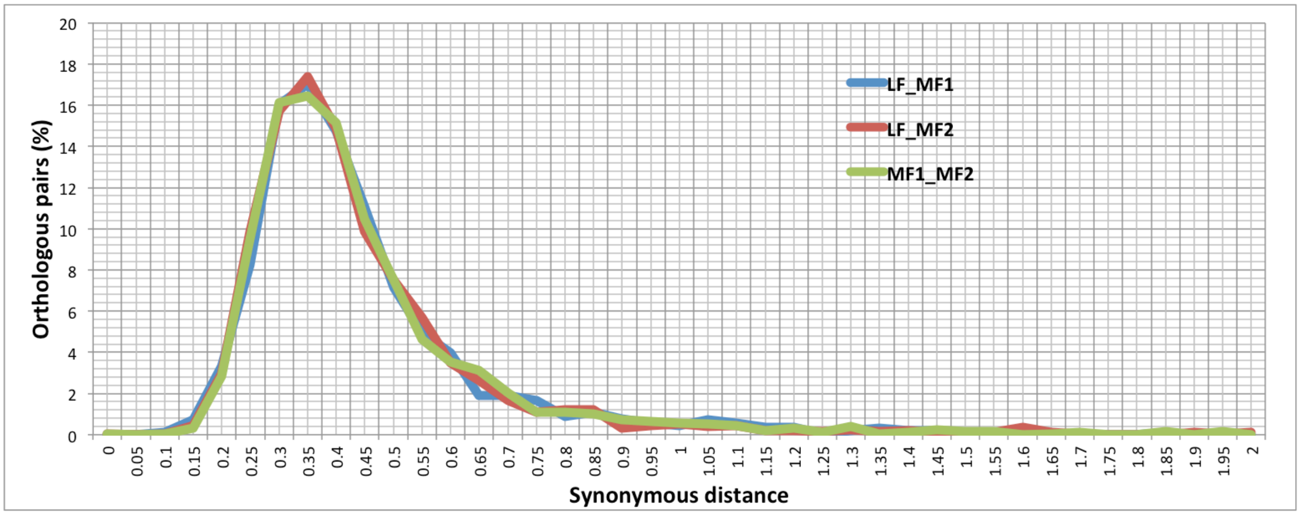

Figure S4: Percentage retention of WGD duplicate gene copies in the *B. rapa* (Br) and *B. oleracea* (Bo) genomes. Number of gene copies are indicated on the x-axis.

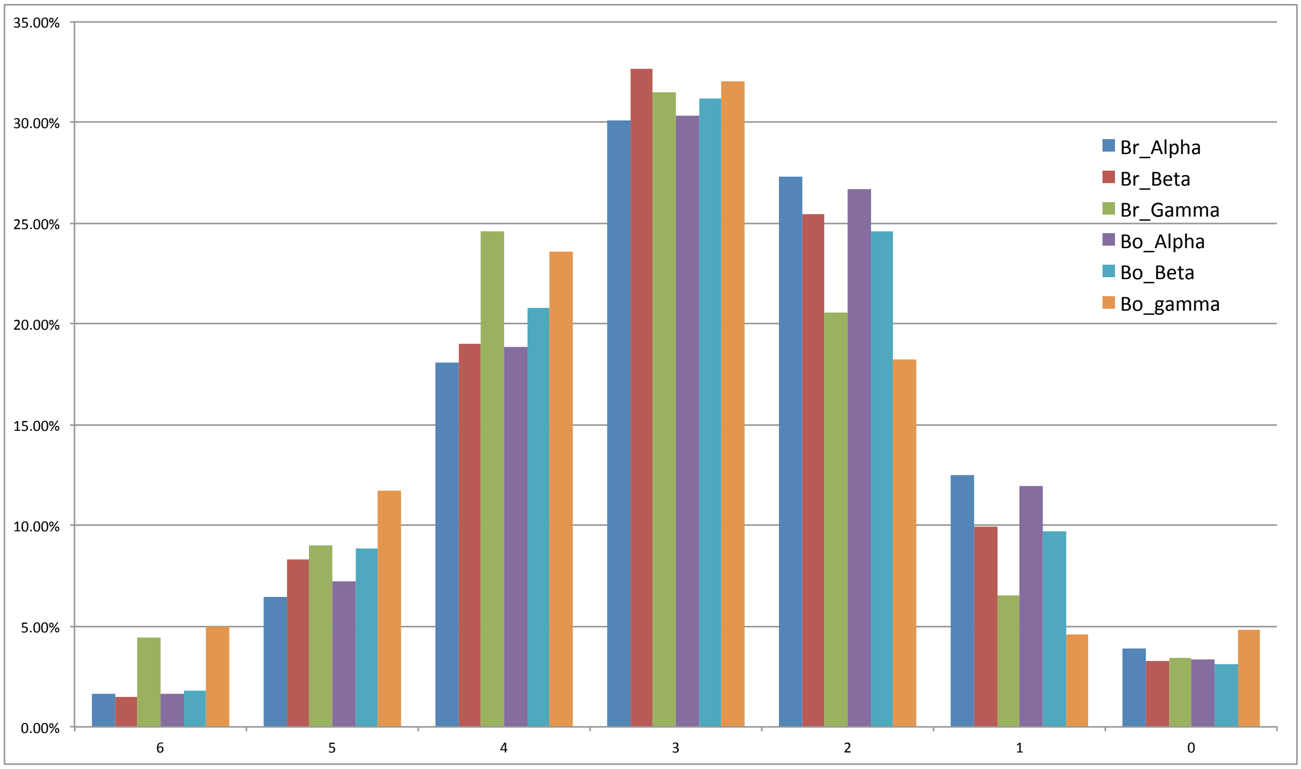

B. oleracea genes corresponding to the α, β and γ paleopolyploidy events were identified based on orthology to the previously established lists of gene pairs representing these three major polyploidy events in *A. thaliana* [1,2]. Ka/Ks ratios were calculated for homologous pairs within each gene-set using the Bioperl script bp_pairwise_kaks.pl [3]. The distributions of the Ka/Ks values for the gene pairs representing alpha, beta and gamma WGD events were compared using the three pairwise Kolmogorov Smirnov (K-S) tests: alpha - beta, beta-gamma, and alpha-gamma. All three tests were significant with $P<2.22e-16$ in each case suggesting that all three distributions are different from each other.

1. Bowers JE, Chapman BA, Rong J, Paterson AH: **Unravelling angiosperm genome evolution by phylogenetic analysis of chromosomal duplication events.** *Nature* 2003, **422:**433-438.
2. Thomas BC, Pedersen B, Freeling M: **Following tetraploidy in an Arabidopsis ancestor, genes were removed preferentially from one homeolog leaving clusters enriched in dose-sensitive genes.** *Genome research* 2006, **16:**934-946.
3. Stajich JE, Block D, Boulez K, Brenner SE, Chervitz SA, Dagdigian C, Fuellen G, Gilbert JG, Korf I, Lapp H, et al: **The Bioperl toolkit: Perl modules for the life sciences.** *Genome research* 2002, **12:**1611-1618.

Figure S5: Cumulative cytosine methylation levels across annotated *B. oleracea* genes .

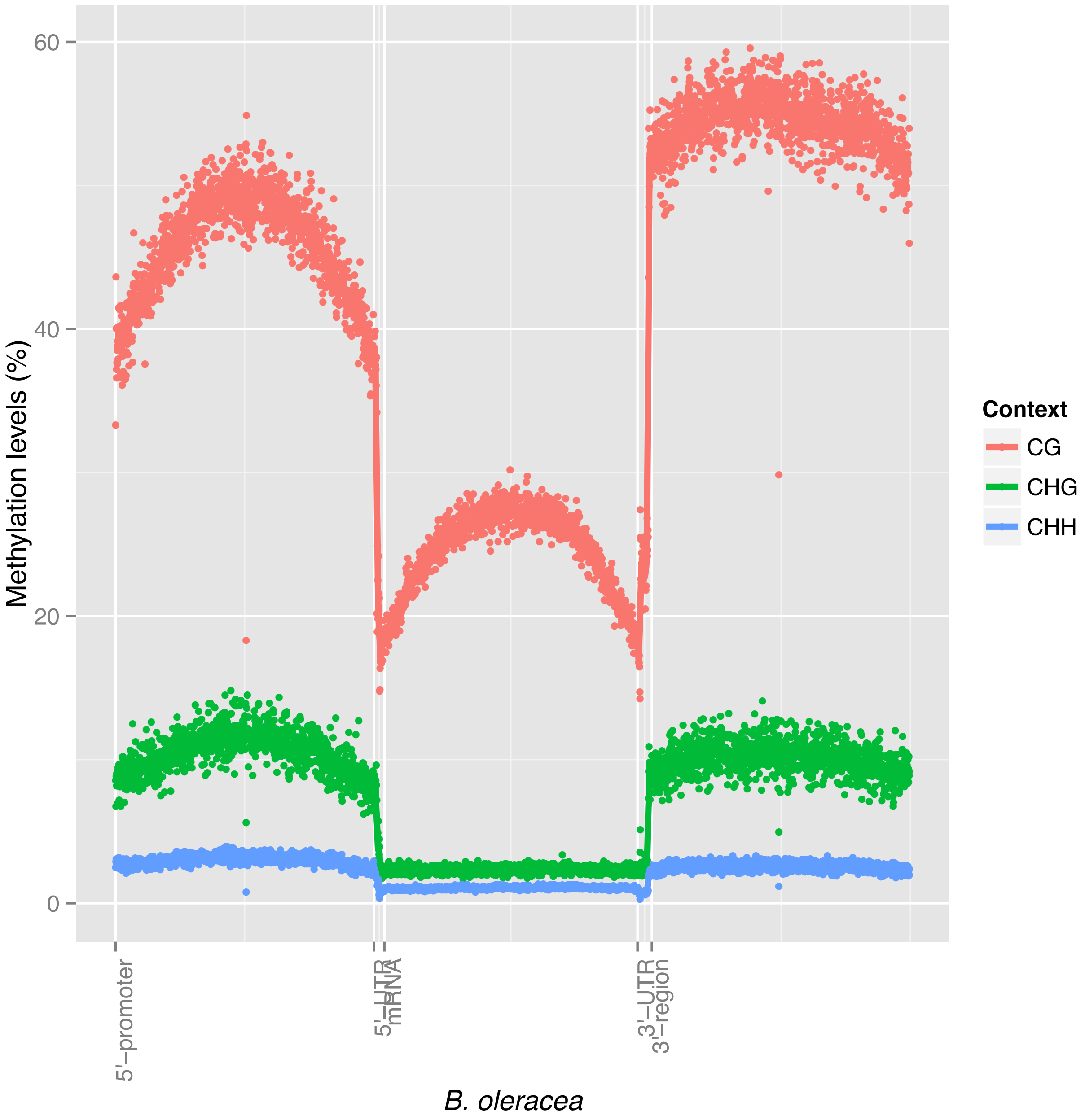

Figure S6: Cytosine methylation levels across specific categories of genes of the *B. oleracea* genome. The mCG (red), mCHG (green) and mCHH (blue) levels are shown based on a sliding window of 500 kb for each a) 5’ promoter; b) 5’ UTR; c) all exons; and d) all introns.

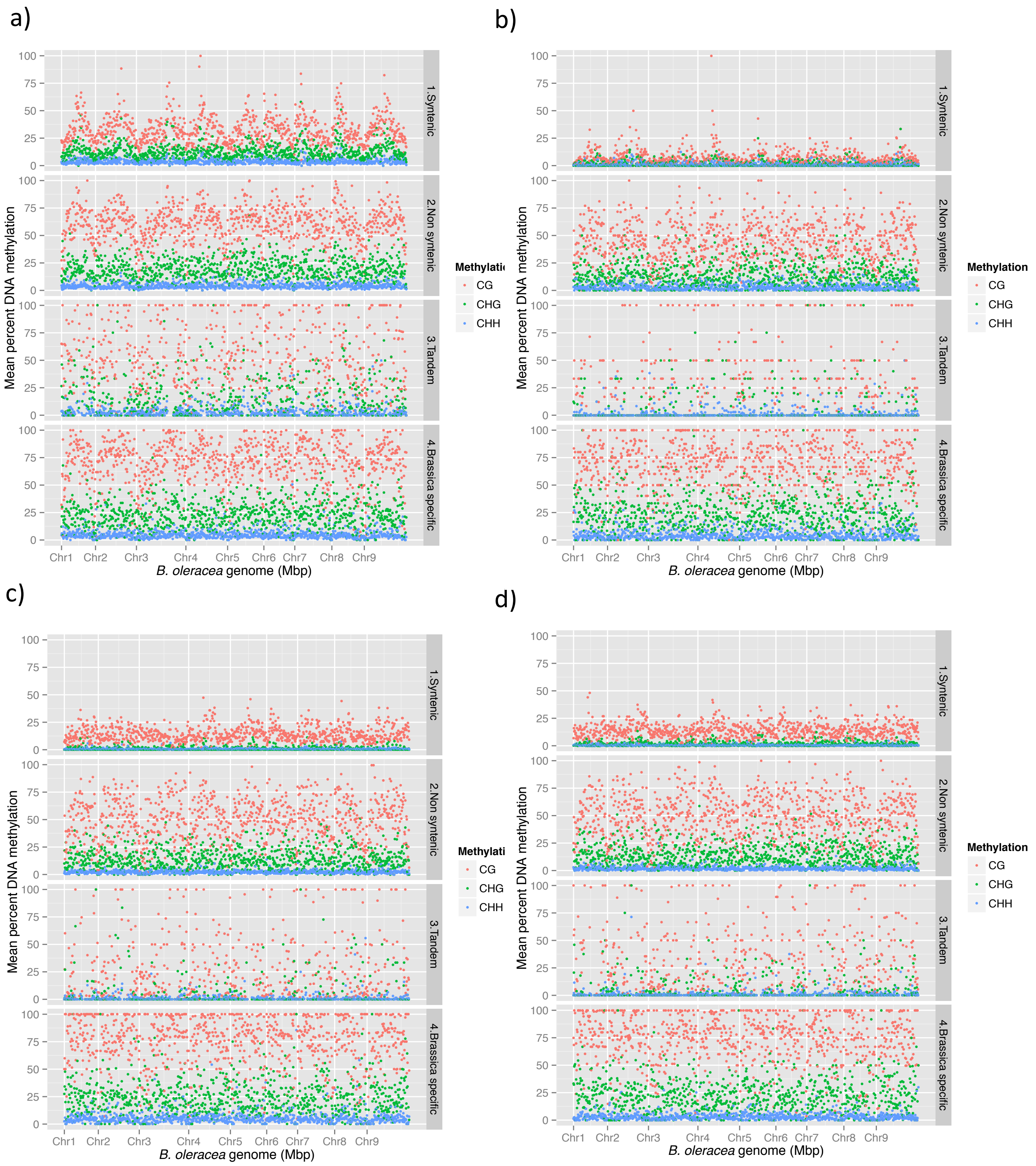

Figure S7: Average gene length (a), exon number (b) and intron number (c) for syntenic, non-syntenic, tandem and *B. oleracea* specific genes

a)

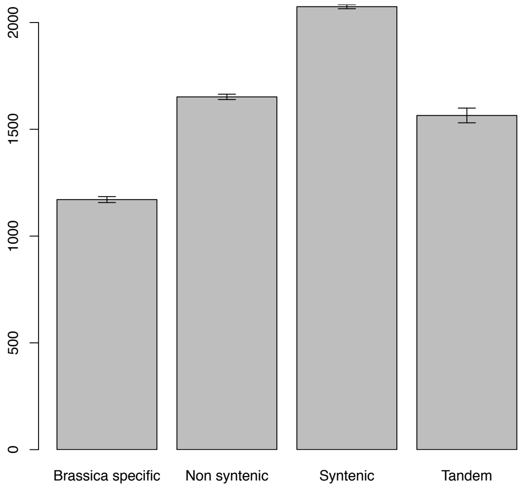

b)

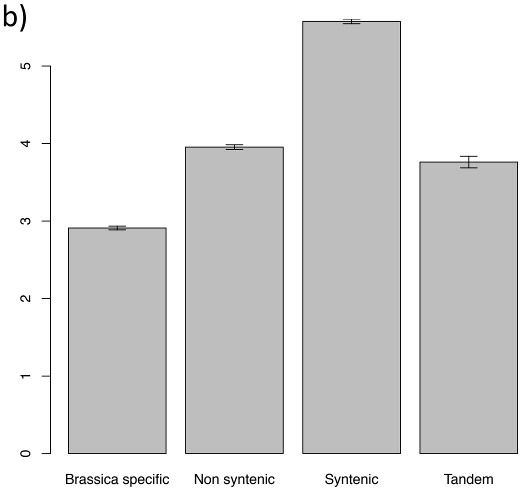

c)

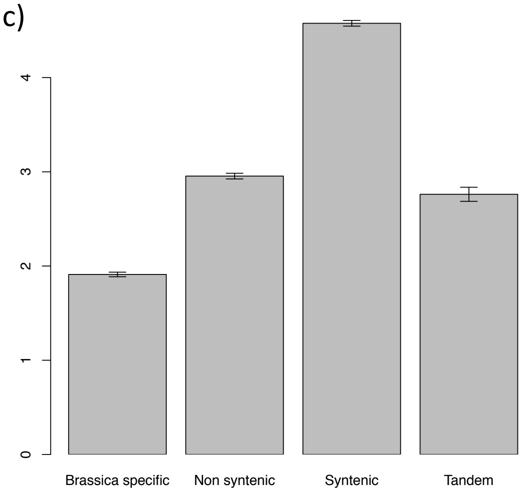

Figure S8: Box plot representation of different levels of a) mCHG and b) mCHH gene body methylation in syntenic genes (along x-axis) with normalized gene expression levels plotted on the y-axis.

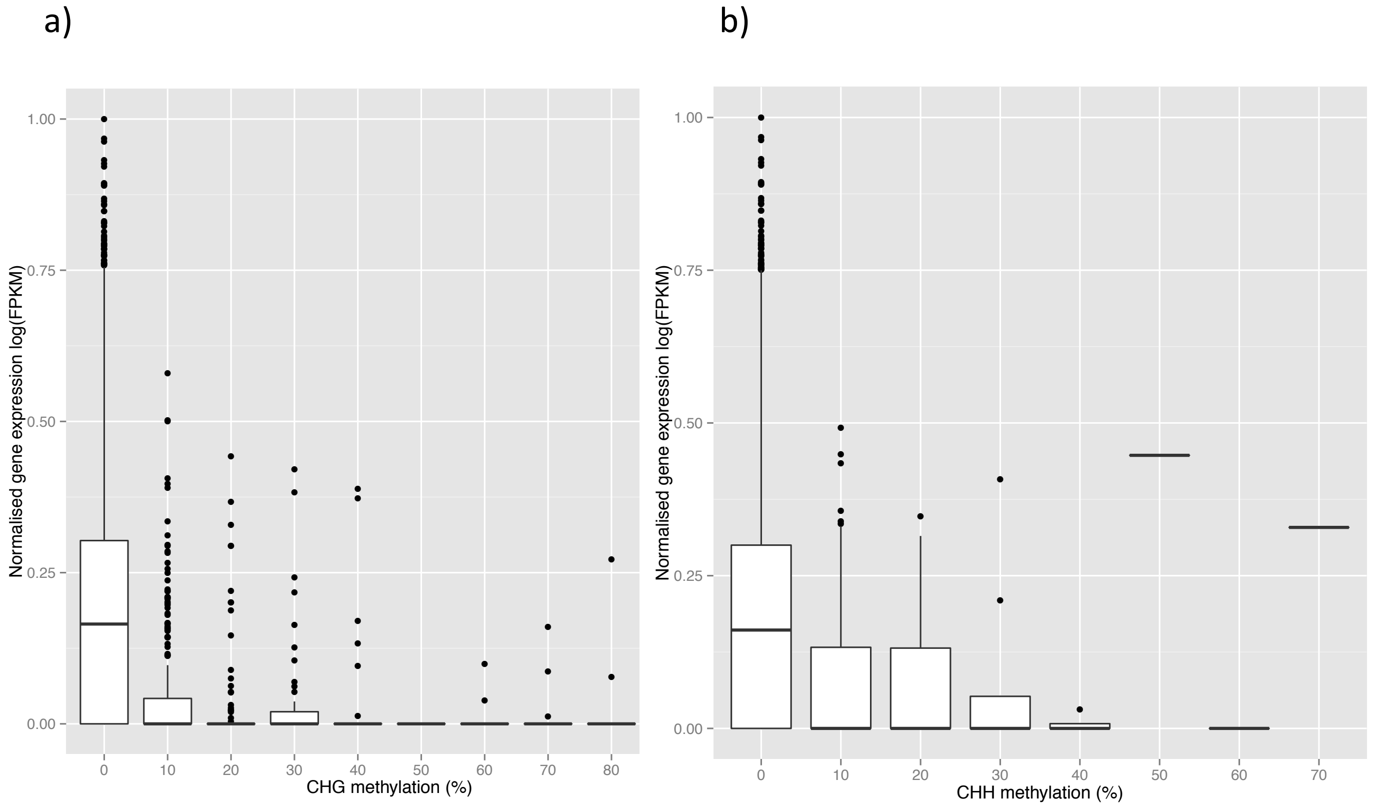

Supplement: Additional file 1: Figure S1 — estimation of genome size based on analysis of 17 bp k‒mer frequency. Figure S2. dotplots representing nucmer alignments of regions of sequence similarity between 18 previously sequenced B. oleracea BACs and the B. oleracea genome sequence. Figure S3.Ks distribution for orthologous gene pairs between (a)A. thaliana and each of the B. oleracea sub‒genomes; and (b) between each of the B. oleracea sub-genomes. Figure S4. percentage retention of WGD duplicate gene copies in the B. rapa (Br) and B. oleracea (Bo) genomes. Figure S5. cumulative cytosine methylation levels across annotated B. oleracea genes. Figure S6. cytosine methylation levels across specific categories of genes of the B. oleracea genome. Figure S7. (a) average gene length, (b) exon number and (c) intron number for syntenic, non‒syntenic, tandem and B. oleracea-specific genes. Figure S8. box plot representation of different levels of (a) mCHG and (b) mCHH gene body methylation in syntenic genes (normalized gene expression levels). [file gb-2014-15-6-r77-S1.pdf]
